# Ganglioside Metabolism and Parkinson's Disease

**DOI:** 10.3389/fnins.2018.00045

**Published:** 2018-02-05

**Authors:** John Forsayeth, Piotr Hadaczek

**Affiliations:** Department of Neurological Surgery, University of California, San Francisco, San Francisco, CA, United States

**Keywords:** glial cell-derived neurotrophic factor, GDNF, B4GALNT1, GM1 ganglioside, alpha-synuclein, Parkinson's disease

## Abstract

Here we advance the hypothesis that Parkinson's disease (PD) is fundamentally a failure of trophic support for specific classes of neurons, primarily catecholaminergic. Evidence from our laboratory provides a framework into which a broad array of findings from many quarters can be integrated into a general theory that offers testable hypotheses to new and established investigators. Mice deficient in the ability to synthesize series-a gangliosides, specifically GM1 ganglioside, develop parkinsonism. We found that this seems to be due to a failure in signaling efficiency by the important catecholaminergic growth factor, GDNF. Interestingly, these mice accumulate alpha-synuclein in nigral neurons. Striatal over-expression of GDNF eliminates these aggregates and also restores normal motor function. These findings bring into question common beliefs about alpha-synuclein pathology and may help us to reinterpret other experimental findings in a new light. The purpose of this article is to provoke new thinking about PD and hopefully encourage younger scientists to explore some of the ideas presented below.

## Introduction

Parkinson's disease (PD) is an anatomically progressive (Del Tredici et al., 2002) neurodegenerative disease that preferentially, but not exclusively, targets catecholaminergic neurons. Although much attention has been focused on dopaminergic neurons in the brain, PD patients suffer a wide array of systemic pathologies that are observable in terms of loss of cardiac sympathetic innervation with consequent orthostatic hypotension (Senard et al., 2001), myenteric neuron atrophy with consequent severe constipation (Stirpe et al., 2016), derangement of olfactory function (Berendse and Ponsen, 2006), loss of cholinergic innervation in pancreas (Gjerloff et al., 2015) and alterations in saccadic eye movement (Buhmann et al., 2015). Abnormalities can also be detected in the skin of PD patients (Gregory and Miller, 2015) and neurovasculature (Mure et al., 2011). In addition, there is strong evidence that synaptic failure rather than cell death is a primary driver of Parkinsonian degeneration (Kordower et al., 2013). Thus, any explanation of where PD pathology arises and what to do about it must advance a common pathological mechanism not limited to specific cellular phenotypes but to more fundamental pathologies. In this article, we propose a comprehensive hypothesis that is worthy of further investigation and, at the very least, advances a remarkable mouse model of the disease that appears to recapitulate spontaneously and progressively many of the major symptoms of the human disease.

## The B4GALNT1 knockout (KO) mouse

**Figure 1:**
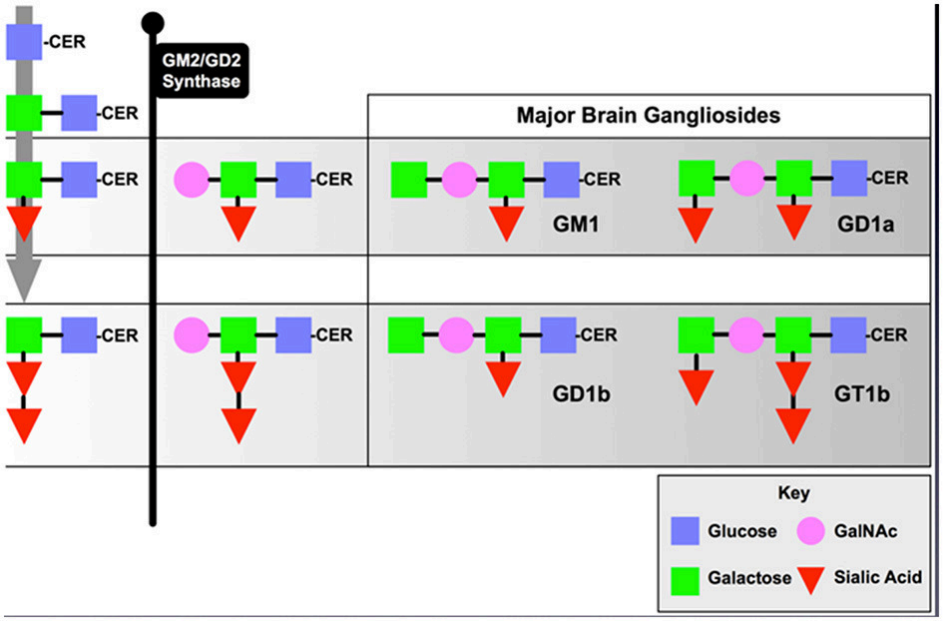
Series-a and Series-b Ganglioside Synthesis Pathway.

The B4GALNT1 gene encodes the enzyme, beta-1,4-N-acetyl-galactosaminyltransferase 1 (GalNac-T), which catalyzes the transfer of N-acetyl galactosamine onto GM3 and GD3 gangliosides resulting in GM2 and GD2 that then undergo further metabolism into GM1 and GD1 (Figure 1). Deletion of this enzyme results in a dramatic loss of series-a and series-b gangliosides in mouse (Sheikh et al., 1999) and human (Harlalka et al., 2013) brain. The KO mice show progressive motor deficits with age (Chiavegatto et al., 2000), a phenomenon attributed at the time to Wallerian degeneration of motor neurons (Sheikh et al., 1999). Recent studies by our groups suggest that the motor deficits observed are due to progressive Parkinsonism as discussed below even though some damage to myelinated neurons has been documented. In contrast, deletion of B4GALNT1 in humans results in severe spastic paraplegia (Harlalka et al., 2013), but this may reflect a much more central role for series-a and series-b gangliosides in myelinated human neurons compared to mice. We have also observed progressive Parkinsonism in B4GALNT1 heterozygotes that, although slower in onset that the homozygotes, is indistinguishable otherwise (Hadaczek et al., 2015). Motor impairment can be observed as early as 35 days of age in terms of grip-strength (Wu et al., 2011) and progressive impairment in a timed-walk and stride-length over several months (Hadaczek et al., 2015). We suspect that the resting tremor documented in these mice (Chiavegatto et al., 2000) is actually a feature of the Parkinsonism rather than peripheral neuropathy, although we have not specifically investigated this feature. The initial observation of Parkinsonism in B4GALNT1 KO mice documented loss of neurons in substantia nigra, a loss that could be substantially ameliorated by dosing animals with the semi-synthetic, brain-permeable GM1 ganglioside analog, LIGA20 (Wu et al., 2011). In addition, nigral accumulation of α-synuclein was also observed and this accumulation could also be reduced by LIGA20 treatment (Wu et al., 2011). This is actually quite a striking observation since a prevalent hypothesis in the field is that accumulation of α-synuclein in PD is due to protein misfolding, a process that one would not expect to be reversible. The ability of LIGA20 to clear α-synuclein aggregates is consistent with the fact that the protein binds GM1 ganglioside, and tends to aggregate in the absence of ganglioside (Martinez et al., 2007). These observations would, however, be of limited value with respect to PD were it not for the fact that reductions in GM1 ganglioside levels in brain tissue from PD patients have been described (Wu et al., 2012; Hadaczek et al., 2015). More importantly, GM1 ganglioside has been explored clinically in controlled and open-label studies (Schneider et al., 1995, 1998, 2010) where modest but significant effects on progression of disease were observed. A limitation of these studies was that they required high daily doses of GM1 (Loading IV dose: 1 g; Daily IM injections BID: 200 mg) for several years. Nevertheless, reduced GM1 ganglioside in mice and humans is associated with Parkinsonian degeneration and supplementing GM1 ganglioside, either directly or via LIGA20, ameliorates disease.

## GDNF signaling and PD

Glial Cell-derived Neurotrophic Factor (GDNF) was isolated from the B49 cell line based on its ability to promote the survival of embryonic DA neurons *in vitro* (Okochi et al., 2000; Nakamura et al., 2002; Taguchi et al., 2017). GDNF was the first identified member of a homologous family of neurotrophic factors related to the basic fibroblast growth factor family. Neurturin (NTN), persephin and artemin were subsequently identified (Zhao et al., 2009). GDNF and its family members act through a receptor signaling system composed of a GPI-linked neurotrophic factor binding subunit and this complex in turn directly activates the C-ret and c-src tyrosine kinases. A multitude of preclinical studies with GDNF protein in an array of rat, mouse and monkey models of PD have demonstrated potent effects of this factor in protecting DA neurons from neurotoxin-induced cell death as well as stimulating regrowth of nigral neurons in primate models of PD (Kozlowski et al., 2000; Eberling et al., 2009; Kells et al., 2010; Lindgren et al., 2012). Clinical experience with recombinant GDNF in PD patients has been mixed. An early Phase 2 study failed to achieve efficacy end-points (Lang et al., 2006) and this has generally been attributed to poor distribution of infused material. A more recent study in which intermittent convection-enhanced delivery (pressurized infusion and reflux-resistant cannula) is employed to cover the majority of the putamen (Belova et al., 2017) seems to be giving more promising results since this approach has completed Phase 2 and is preparing to move into Phase 3 (see Medgenesis.com). Poor efficacy of a GDNF analog encoded within an AAV2 vector, Neurturin, was also reported (Marks et al., 2010). Our view is that poor distribution of infused factor or gene therapy vector is the primary driver of poor efficacy. In our current gene transfer study being conducted at NIH Clinical Center (Bethesda, MD) and at our institution, use of MRI guidance and relatively high infusion volumes (Richardson et al., 2011) to ensure adequate coverage of the putamen and caudate with AAV2-GDNF should provide a definitive answer to the utility of GDNF in Parkinson's disease in line with encouraging data from stable MPTP-lesioned nonhuman primates (Kells et al., 2010).

It is important, in the present context, to consider the molecular biology of GDNF function. First, GDNF itself reaches peak expression prenatally (Hellmich et al., 1996) where it presumably aids in the upregulation of dopaminergic function, a phenomenon that we have noted with over-expression of GDNF in partially lesioned MPTP monkeys (Kells et al., 2010). Postnatally, however, GDNF expression measured by ELISA falls to very low levels indeed (Collier et al., 2005; Johnston et al., 2009). One explanation for this change is that pro-GDNF exists as two isoforms, (alpha)pro-GDNF and (beta)pro-GDNF in which alternative cleavable pro-peptides direct GDNF into either a constitutive (alpha) or vesicular (beta) pathway (Lonka-Nevalaita et al., 2010). In the striatum, GDNF-positive cells have been identified in the mouse as parvalbumin-positive (PV+) interneurons, and to a smaller extent cholinergic interneurons (Hidalgo-Figueroa et al., 2012). Although these neurons represent < 1% of all striatal neurons in the mouse, they appear to be very broadly and evenly distributed. Taken together, these observations suggest that GDNF undergoes a developmental switch, whereby constitutive embryonic secretion broadly upregulates catecholaminergic function and promotes survival of this class of neurons, and then is followed by postnatal restriction to dense-core vesicles that respond to extracellular stimuli by local release of GDNF. We surmise that the locally released GDNF acts to upregulate presynaptic dopaminergic function depending on a variety of molecular cues. In this way, striatal interneurons may play an important coordinating role in striatal plasticity. If so, then any pathology that injures this mechanism might be expected to result in down-regulation of dopaminergic function, starting with degeneration of dopaminergic synapses, a phenomenon seen in normal aging (Johnston et al., 2009) and PD (Kordower et al., 2013).

GDNF is primarily recognized by an extracellular GPI-linked receptor subunit (Jing et al., 1996; Treanor et al., 1996) that is localized to lipid rafts (Tansey et al., 2000; Pierchala et al., 2006). Binding of GDNF to its receptor, GFRα1, recruits a tyrosine kinase, RET, into rafts where it undergoes autophosphorylation, a process that protects it from proteasomal degradation (Pierchala et al., 2006). RET is not the only tyrosine kinase activated by GDNF. Neuronal *src*, a splice variant with an N-terminal palmitoylation site also targets the enzyme to lipid rafts (Mukherjee et al., 2003), where it cooperates with RET (Encinas et al., 2001) to regulate downstream signaling, but can also be activated directly by GDNF in a RET-independent manner (Poteryaev et al., 1999). This pleiotropic signaling mechanism also involves at least one other membrane-bound tyrosine kinase, *c-abl*, which is activated by *src*-dependent phosphorylation (Plattner et al., 1999). It is important to note that lipid rafts are organizing structures on cell surfaces and organelles (Lingwood and Simons, 2010). They are highly enriched in cholesterol and can be either short-lived or very stable. Functionally, they allow subsets of membrane proteins to interact in a coordinated way and they play a central role in extracellular signaling by a variety of molecules including neurotrophins like GDNF. Gangliosides are enriched in lipid rafts because of their long alkyl chains and oligosaccharide head-groups, providing membrane surface components through which proteins can interact.

**Figure 2:**
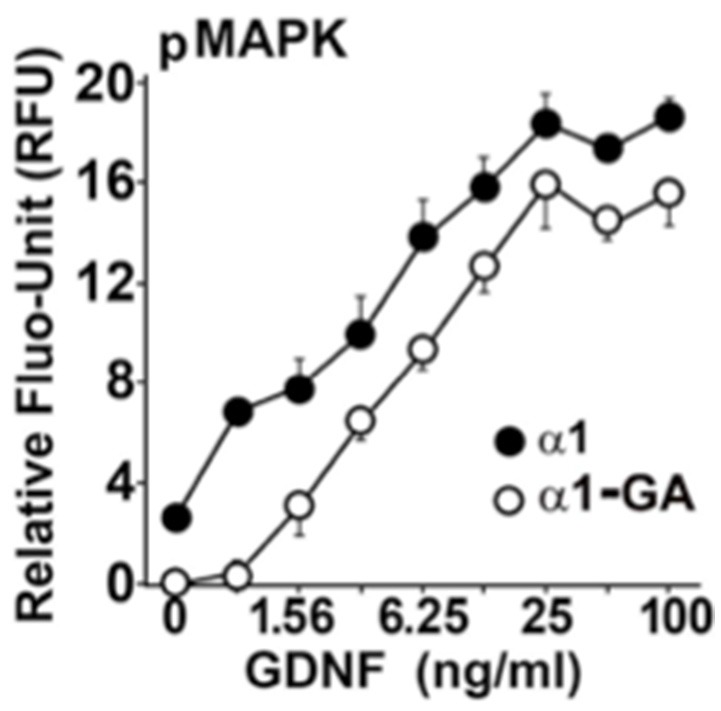
GM1 regulation of GDNF signaling in modified mouse Neuro2a cells Neuro2a cells modified by over-expression of hGFRα1 (α1) (Crowder et al., 2004), and α1-GA cells with approximately 50% reduced expression of GM2/GD2 synthase. Cells were stimulated with different concentrations of GDNF. The level of MAPK (Erk1/2) phosphorylation was measured by an in-cell ELISA as described (Hadaczek et al., 2015).

We hypothesized that the Parkinsonism, evinced by both homozygous and heterozygous B4GALNT1 mice, might be due to a GM1 ganglioside requirement by the GDNF receptor for efficient signaling. In a mouse Neuro2a cell line, stably over-expressing human GFRα1 (Hadaczek et al., 2015), human GDNF strongly stimulated phosphorylation of MAP kinase, making it a useful marker of GDNF bioactivity (Figure 2). In contrast, suppression of B4GALNT1 expression with shRNA shifted the GDNF dose-response curve sharply to the right. Consistent with this, we found that GFRα1, RET, and GM1 ganglioside forms a stable membrane complex in the substantia nigra of wild-type mice, but not KO mice. Incidentally, other series-a and series-b gangliosides are not present in this complex. On this basis, we argue that even modest declines in GM1 ganglioside levels should inhibit trophic support in dopaminergic neurons.

## GM1 ganglioside and α-synuclein

A prominent hypothesis posits that α-synuclein misfolding is a central cause of PD. Although it is true that mutations in α-synuclein and triplication of its gene (SNCA) are causative (Polymeropoulos et al., 1997; Singleton et al., 2003), they do not necessarily trigger disease onset significantly in advance of the onset of idiopathic PD (>60 years of age), unlike what is seen with Parkin-mediated PD (< 40 years of age). This suggests that synuclein-mediated PD is enabled by other age-dependent and environmental cellular changes. In our view, age-dependent depletion of GM1 ganglioside is a prime suspect.

**Figure 3:**
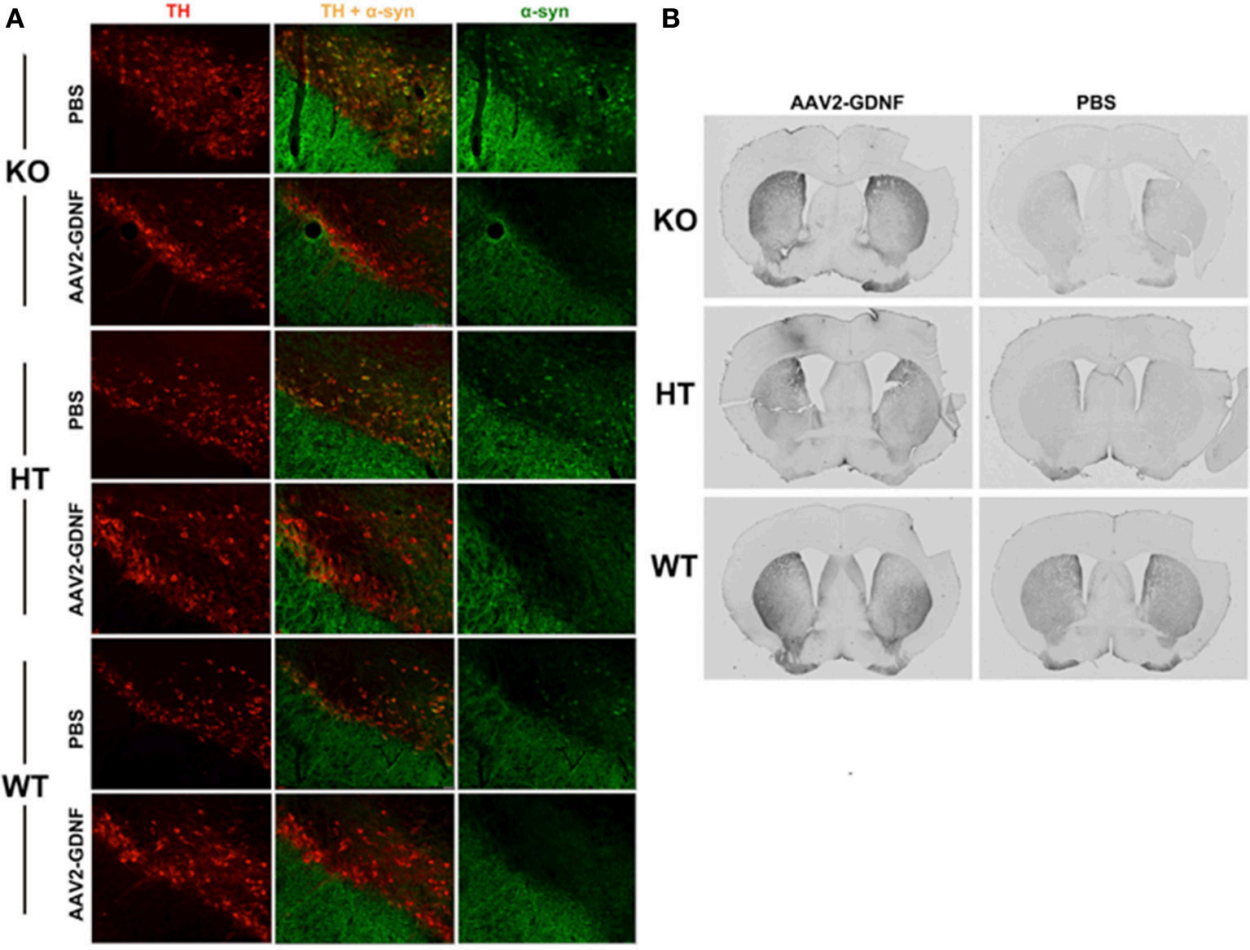
Elimination of α-synuclein and restoration of TH expression in nigral neurons of GM1-deficient mice with AAV2-GDNF treatment. **(A)** Representative double immunostained brain sections from mice of the three genotypes, employing antibodies against α-synuclein and tyrosine hydroxylase (TH). Alpha-synuclein expression in HT mice was enhanced compared to WT, and more so for KO mice. These elevated levels of α-synuclein (α-syn) were significantly reduced by treatment with striatal infusion of AAV2-GDNF versus control PBS. It should also be noted that mice of approximately 12 months of age spontaneously accumulate α-synuclein. Striatal over-expression of GDNF eliminated these aggregates. **(B)** Representative images showing immunohistochemical staining of TH^+^ neurons in mouse striatal sections. AAV2-GDNF treatment restored significant expression of TH in both HT and KO mice compared to PBS. Reprinted from Hadaczek et al. (2015). Copyright 2015, with permission from Elsevier.

In accord with this thinking is our finding that α-synuclein accumulated as aggregates in the substantia nigra pars compacta of B4GALNT KO mice as well as heterozygotes (Figure 3) and these aggregates could be completely eliminated by striatal over-expression of GDNF, a treatment that also eliminated motor deficits (Hadaczek et al., 2015). This suggests that (a) depletion of GM1 ganglioside drives both α-synuclein accumulation/aggregation and GDNF resistance/Parkinsonism, and (b) α-synuclein aggregates can be eliminated by local concentrations of GDNF sufficiently high to overcome the impairment of GDNF signaling caused by GM1 ganglioside depletion. Strikingly, the same phenomenon could be seen in aged normal mice, which spontaneously accumulated nigral α-synuclein. GDNF over-expression not only eliminated aggregates but also improved motor function in GDNF-treated animals (Hadaczek et al., 2015). On this basis, we argue that α-synuclein accumulation/aggregation is a biomarker for impaired trophic signaling and, in some cases, reduced GM1 ganglioside levels.

That α-synuclein is not particularly neurotoxic is supported by experiments in which wild-type human α-synuclein was highly over-expressed in transgenic mice with modest evidence of neuronal toxicity (Masliah et al., 2000) that most closely resembled Lewy Body disease rather than PD. Subsequent work by other investigators has only served to establish more firmly that α-synuclein demonstrates neurotoxicity only at the very highest levels of expression achievable and does not drive the development of cardinal symptoms of PD (Terzioglu and Galter, 2008). We would argue that high levels of α-synuclein, regardless of the delivery method (Recasens et al., 2017), are likely to cause degenerative effects precisely because it is a GM1 ganglioside- and cholesterol-binding protein that also targets lipid rafts. If that critical fact is ignored, then in our view merely over-expressing α-synuclein results in essentially phenomenological reporting.

Our thesis is that causative mutations in α-synuclein can only really be understood if the normal functions of the protein in question are explicated. It is, of course, formally possible that familial mutations confer toxic activity unrelated to the normal function of the protein. Nevertheless, it is a core principle of biology that any understanding of protein function must ultimately be derived from an understanding of conserved motifs within its primary sequence (Aitken, 1999). For a small protein of only 140 amino acids, α-synuclein is richly endowed with such motifs and most of them have been studied individually from various perspectives. However, there appears to have been little attempt to develop a coordinated picture of how individual motifs direct an overall mechanism of action or function, whatever that may be.

**Figure 4:**
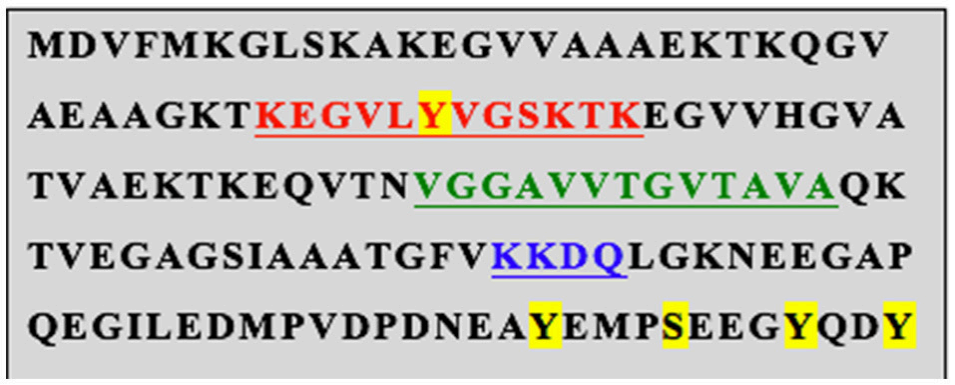
Conserved Motifs within human α-synuclein. Putative phosphorylation sites are highlighted in yellow. The red text indicates a glycosphingolipid (GM1) binding site within which resides a cholesterol binding motif (VLYVG). Further down is another non-equivalent cholesterol-binding motif shown in green. Finally, the blue text indicates the LAMP2a consensus. The same motifs are present in the mouse homolog.

Regarding associated motifs, it is important to note that α-synuclein (Figure 4) is a ganglioside-binding protein (Martinez et al., 2007; Fantini and Yahi, 2011) that forms fibrils in the absence of GM1 ganglioside (Martinez et al., 2007; Fantini and Yahi, 2011). This specific motif (34-KEGVLYVGSKTK-45) is also conserved in a number of other proteins, such as prion protein and β-amyloid (Amyloid beta peptide) (Yahi and Fantini, 2014). It is interesting that some α-synuclein mutations that cause PD flank this domain (e.g., A30P and E46K) and the A30P mutation has been reported not to bind GM1 ganglioside (Martinez et al., 2007). Structural studies have shown that this region of α-synuclein is helical. Thus, any mutation that disrupts this helicity is likely to damage GM1 ganglioside binding, a feature that we argue is central to the protein's biological function (Bisaglia et al., 2006; Dettmer et al., 2015). A remarkable feature of this domain is the presence of a highly conserved central tyrosine residue (Y39) that appears to be important for the ability of the protein to insert into membranes (Fantini and Yahi, 2011). This residue is phosphorylated by *c-abl in vitro* (Dikiy et al., 2016) and there is evidence that this also occurs *in vivo* (Mahul-Mellier et al., 2014). Associated with lipid rafts, *c-abl* is actuated by various growth factors via upstream activation of *c-src* (Plattner et al., 1999). This link between growth factor signaling and α-synuclein phosphorylation is intriguing, especially in view of our observation that over-expressed GDNF in mouse striatum eliminates α-synuclein aggregates in GM1 ganglioside-deficient mice and even in normal aged mice (Hadaczek et al., 2015).

In addition, Sulzer and colleagues (Cuervo et al., 2004) showed that α-synuclein is recognized by the lysosomal membrane protein LAMP2a via the sequence, VKKDQ, and this mediates entry into the lumen of the lysosome. Thus, mutating VKKDQ to VKKAA abolished LAMP2a binding and intra-luminal transport of α-synuclein. These investigators focused on this process as a mechanism of autophagic degradation of the protein. However, an interesting study of cells derived from a patient with Danon disease, of which LAMP2a deficiency is a central feature, reported that α-synuclein in patient-derived lymphoblastoid cells was not elevated, challenging the idea that LAMP2a function primarily mediates α-synuclein autophagy under normal conditions (Sanchez-Lanzas et al., 2016). Interestingly, they showed that, although the A30P and A53T mutants bound to LAMP2a at least as well as wild-type α-synuclein, they were not translocated efficiently into the lysosomal lumen, thereby leading to accumulation of cytoplasmic α-synuclein and inhibition of autophagy of other proteins such as GAPDH.

The Fantini laboratory identified 2 non-equivalent cholesterol-binding domains within α-synuclein (Fantini and Yahi, 2013). The first is contained within the GM1 ganglioside motif and we speculate that, given the cholesterol-rich environment of lipid rafts, this motif (VLVYVGSK) might enable competitive displacement of GM1 ganglioside from the protein. The downstream cholesterol-binding site (67-GGAVVTGVTAVA-78), a so-called CRAC domain (Fantini et al., 2011), is more complicated and is argued to mediate conformational changes when α-synuclein encounters cholesterol-rich membranes. Since α-synuclein selectively binds to cholesterol-rich lipid rafts on the surface of organelles and cell membranes (Fortin et al., 2004; Zabrocki et al., 2008), it seems reasonable to infer that it functions as a GM1 ganglioside binding protein whose primary role in neurons is to deliver this important glycosphingolipid to lipid rafts. This hypothesis might explain the enhanced PD risk in Gaucher disease heterozygotes. A 50% reduction in lysosomal glucocerebrosidase activity could significantly perturb the kinds of glycolipids available for binding to α-synuclein and perhaps deliver poorly functional glycosphingolipids to lipid rafts throughout the cell. A hierarchy of binding affinities of α-synuclein for various glycolipids has been described (Fantini and Yahi, 2011). Recently, Mazzulli et al showed that iPSC-derived neuronal cells from Gaucher heterozygotes displayed increased α-synuclein aggregation (Mazzulli et al., 2011). These data suggest that modest alterations in lysosomal function and content can change glycolipid function and the behavior of α-synuclein, a concept supported by recent data (Taguchi et al., 2017).

A central problem with ganglioside metabolism in neurons is that these gangliosides are synthesized within the ER/Golgi or endocytosed from the extracellular milieu. How then are these molecules delivered efficiently to the cell's lipid rafts? Our view is that lysosomes provide the primary source of GM1 ganglioside for this purpose. This would also explain the mechanism described by Cuervo et al. (2004), although they argued that the primary reason for this phenomenon was chaperone-mediated autophagy (CMA), whereas other investigators have argued in favor of proteasomal degradation after ubiquitination. We suggest an alternative hypothesis, that this, the α-synuclein transport step into the lysosomal lumen is followed by binding of luminal GM1 ganglioside, either synthesized *de novo* or recycled by autophagy. This combination enables non-transporter-mediated escape of the GM1 α-synuclein moiety from lysosomes and makes the complex available to bind to cholesterol-rich lipid rafts within the cell. We suggest that, upon binding to a lipid raft, α-synuclein is induced to insert the GM1 into the membrane, through a cooperative interaction between cholesterol and GM1 within the GM1 binding site. This mechanism would explain the delivery and release of GM1 into the lipid raft structure but does not resolve the problem of α-synuclein removal from the lipid raft. For the latter, we hypothesize that this might be accomplished by phosphorylation of α-synuclein at Y39 (Mahul-Mellier et al., 2014), possibly by *c-abl* (Dikiy et al., 2016). Phosphorylation would block the binding of both GM1 and cholesterol, and the additional negatively charged phosphoryl group could contribute to ejection process from the membrane. In addition, the C-terminal region contains another three putative tyrosine phosphorylation sites (Nakamura et al., 2002; Mahul-Mellier et al., 2014) and a single serine phosphorylation site at S129 (Okochi et al., 2000). It is not clear whether these sites also play a role in ejection from the lipid raft, but it has been suggested that tyrosine phosphorylation by *c-abl*, a lipid raft resident (Zhao et al., 2009), perhaps at one or both of these sites, may also mediate proteasomal degradation of α-synuclein (Mahul-Mellier et al., 2014).

If, as we suggest, α-synuclein plays a key role in delivering GM1 ganglioside to lipid rafts and this is important for trophic support, why does deletion of SNCA in mice not cause PD? First of all, deletion of α-synuclein in mice causes significant alterations in brain lipid metabolism (Castagnet et al., 2005; Golovko et al., 2005; Barcelo-Coblijn et al., 2007). Second, a closely related homolog, β-synuclein, is co-expressed with α-synuclein *in vivo* and might compensate for the deletion (Israeli and Sharon, 2009) If so, a double deletion of both α- and β-synuclein should cause dopaminergic neuron degeneration and indeed a modest (20%) decline in brain dopamine was seen in such mice (Chandra et al., 2004). However, these mice were studied for only the first 3–5 weeks of life. As we have observed above, GM1 ganglioside deficiency has significant degenerative effects on dopaminergic in substantia nigra when mice are over 6 months of age.

This hypothesis has important implications for both PD and Lewy Body Dementia. If, as we suppose, failure of trophic signaling in lipid rafts leads to depressed clearance of α-synuclein from these structure, then accumulation of the protein might drive structural changes sufficient to activate an innate immune response (Block et al., 2007). Accumulation of aggregated α-synuclein in cell surface rafts would be a mechanism by which microglia are alerted to these aberrant moieties (Zhang et al., 2005) and might explain the presence of nitrated α-synuclein in Lewy Bodies if not the genesis of Lewy Bodies themselves (Danielson and Andersen, 2008). The fact that Tyrosine-39 in α-synuclein is preferentially nitrated under conditions of oxidative stress is noteworthy (Danielson et al., 2009).

## Conclusion

In this review, we present a new hypothesis about the origin of PD designed to provoke discussion and hopefully more research. The theory that age-dependent GM1 ganglioside deficiency is one trigger for idiopathic PD is attractive not only because it is supported by clinical and animal data but also because it recognizes that there are many potential mechanisms besides GM1 ganglioside loss that could drive trophic signaling failure that in turn would lead to accumulation of α-synuclein in neuronal membranes and activation of innate immunity. In that sense, it is inclusive of many other related cellular and genetic phenomena not discussed here.

## Author contributions

JF: wrote the article; PH: provided data and helped revise the manuscript.

### Conflict of interest statement

The authors declare that the research was conducted in the absence of any commercial or financial relationships that could be construed as a potential conflict of interest.

## References

[B1] Aitken A. (1999). Protein consensus sequence motifs. Mol. Biotechnol. 12, 241–253. 10.1385/MB:12:3:241 10631681

[B2] Barcelo-Coblijn G. Golovko M. Y. Weinhofer I. Berger J. Murphy E. J. (2007). Brain neutral lipids mass is increased in α-synuclein gene-ablated mice. J. Neurochem. 101, 132–141. 10.1111/j.1471-4159.2006.04348.x 17250686

[B3] Belova E. Shaffer C. L. Trapa P. E. (2017). Insights from mathematical modeling for convection-enhanced intraputamenal delivery of GDNF. Med. Biol. Eng. Comput. 55, 2069–2077. 10.1007/s11517-017-1650-x 28493093 PMC5680405

[B4] Berendse H. W. Ponsen M. M. (2006). Detection of preclinical Parkinson's disease along the olfactory trac(t). J. Neural Transm. Suppl. 70, 321–325. 10.1007/978-3-211-45295-0_48 17017547

[B5] Bisaglia M. Schievano E. Caporale A. Peggion E. Mammi S. (2006). The 11-mer repeats of human α-synuclein in vesicle interactions and lipid composition discrimination: a cooperative role. Biopolymers 84, 310–316. 10.1002/bip.20440 16411187

[B6] Block M. L. Zecca L. Hong J. S. (2007). Microglia-mediated neurotoxicity: uncovering the molecular mechanisms. Nat. Rev. Neurosci. 8, 57–69. 10.1038/nrn2038 17180163

[B7] Buhmann C. Kraft S. Hinkelmann K. Krause S. Gerloff C. Zangemeister W. H. (2015). Visual attention and saccadic oculomotor control in Parkinson's disease. Eur. Neurol. 73, 283–293. 10.1159/000381335 25925289

[B8] Castagnet P. I. Golovko M. Y. Barcelo-Coblijn G. C. Nussbaum R. L. Murphy E. J. (2005). Fatty acid incorporation is decreased in astrocytes cultured from α-synuclein gene-ablated mice. J. Neurochem. 94, 839–849. 10.1111/j.1471-4159.2005.03247.x 16033426

[B9] Chandra S. Fornai F. Kwon H. B. Yazdani U. Atasoy D. Liu X.. (2004). Double-knockout mice for α- and β-synucleins: effect on synaptic functions. Proc. Natl. Acad. Sci. U.S.A. 101, 14966–14971. 10.1073/pnas.0406283101 15465911 PMC522043

[B10] Chiavegatto S. Sun J. Nelson R. J. Schnaar R. L. (2000). A functional role for complex gangliosides: motor deficits in GM2/GD2 synthase knockout mice. Exp. Neurol. 166, 227–234. 10.1006/exnr.2000.7504 11085888

[B11] Collier T. J. Dung Ling Z. Carvey P. M. Fletcher-Turner A. Yurek D. M. Sladek J. R. Jr.. (2005). Striatal trophic factor activity in aging monkeys with unilateral MPTP-induced parkinsonism. Exp. Neurol. 191(Suppl. 1), S60–S67. 10.1016/j.expneurol.2004.08.018 15629762

[B12] Crowder R. J. Enomoto H. Yang M. Johnson E. M. Jr. Milbrandt J. (2004). Dok-6, a Novel p62 Dok family member, promotes Ret-mediated neurite outgrowth. J. Biol. Chem. 279, 42072–42081. 10.1074/jbc.M403726200 15286081

[B13] Cuervo A. M. Stefanis L. Fredenburg R. Lansbury P. T. Sulzer D. (2004). Impaired degradation of mutant α-synuclein by chaperone-mediated autophagy. Science 305, 1292–1295. 10.1126/science.1101738 15333840

[B14] Danielson S. R. Andersen J. K. (2008). Oxidative and nitrative protein modifications in Parkinson's disease. Free Radic. Biol. Med. 44, 1787–1794. 10.1016/j.freeradbiomed.2008.03.005 18395015 PMC2422863

[B15] Danielson S. R. Held J. M. Schilling B. Oo M. Gibson B. W. Andersen J. K. (2009). Preferentially increased nitration of α-synuclein at tyrosine-39 in a cellular oxidative model of Parkinson's disease. Anal. Chem. 81, 7823–7828. 10.1021/ac901176t 19697948 PMC2748813

[B16] Del Tredici K. Rub U. De Vos R. A. Bohl J. R. Braak H. (2002). Where does parkinson disease pathology begin in the brain? J. Neuropathol. Exp. Neurol. 61, 413–426. 10.1093/jnen/61.5.413 12030260

[B17] Dettmer U. Newman A. J. Soldner F. Luth E. S. Kim N. C. von Saucken V. E. (2015). Parkinson-causing α-synuclein missense mutations shift native tetramers to monomers as a mechanism for disease initiation. Nat. Commun. 6:7314 10.1038/ncomms8314 26076669 PMC4490410

[B18] Dikiy I. Fauvet B. Jovicic A. Mahul-Mellier A. L. Desobry C. El-Turk F.. (2016). Semisynthetic and *in vitro* phosphorylation of α-synuclein at Y39 promotes functional partly helical membrane-bound states resembling those induced by PD mutations. ACS Chem. Biol. 11, 2428–2437. 10.1021/acschembio.6b00539 27356045 PMC5240779

[B19] Eberling J. L. Kells A. P. Pivirotto P. Beyer J. Bringas J. Federoff H. J.. (2009). Functional effects of AAV2-GDNF on the dopaminergic nigrostriatal pathway in parkinsonian rhesus monkeys. Hum. Gene Ther. 20, 511–518. 10.1089/hum.2008.201 19254173 PMC2725183

[B20] Encinas M. Tansey M. G. Tsui-Pierchala B. A. Comella J. X. Milbrandt J. Johnson E. M.. (2001). c-Src is required for glial cell line-derived neurotrophic factor (GDNF) family ligand-mediated neuronal survival via a phosphatidylinositol-3 kinase (PI-3K)-dependent pathway. J Neurosci. 21, 1464–1472. 11222636 10.1523/JNEUROSCI.21-05-01464.2001 PMC6762937

[B21] Fantini J. Carlus D. Yahi N. (2011). The fusogenic tilted peptide (67-78) of α-synuclein is a cholesterol binding domain. Biochim. Biophys. Acta 1808, 2343–2351. 10.1016/j.bbamem.2011.06.017 21756873

[B22] Fantini J. Yahi N. (2011). Molecular basis for the glycosphingolipid-binding specificity of α-synuclein: key role of tyrosine 39 in membrane insertion. J. Mol. Biol. 408, 654–669. 10.1016/j.jmb.2011.03.009 21396938

[B23] Fantini J. Yahi N. (2013). The driving force of α-synuclein insertion and amyloid channel formation in the plasma membrane of neural cells: key role of ganglioside- and cholesterol-binding domains. Adv. Exp. Med. Biol. 991, 15–26. 10.1007/978-94-007-6331-9_2 23775688

[B24] Fortin D. L. Troyer M. D. Nakamura K. Kubo S. Anthony M. D. Edwards R. H. (2004). Lipid rafts mediate the synaptic localization of α-synuclein. J. Neurosci. 24, 6715–6723. 10.1523/JNEUROSCI.1594-04.2004 15282274 PMC6729723

[B25] Gjerloff T. Fedorova T. Knudsen K. Munk O. L. Nahimi A. Jacobsen S.. (2015). Imaging acetylcholinesterase density in peripheral organs in Parkinson's disease with 11C-donepezil PET. Brain 138(Pt 3), 653–663. 10.1093/brain/awu369 25539902 PMC4408425

[B26] Golovko M. Y. Faergeman N. J. Cole N. B. Castagnet P. I. Nussbaum R. L. Murphy E. J. (2005). α-synuclein gene deletion decreases brain palmitate uptake and alters the palmitate metabolism in the absence of α-synuclein palmitate binding. Biochemistry 44, 8251–8259. 10.1021/bi0502137 15938614

[B27] Gregory R. Miller S. (2015). Parkinson's disease and the skin. Pract. Neurol. 15, 246–249. 10.1136/practneurol-2015-001107 25862733

[B28] Hadaczek P. Wu G. Sharma N. Ciesielska A. Bankiewicz K. Davidow A. L.. (2015). GDNF signaling implemented by GM1 ganglioside; failure in Parkinson's disease and GM1-deficient murine model. Exp. Neurol. 263, 177–189. 10.1016/j.expneurol.2014.10.010 25448159

[B29] Harlalka G. V. Lehman A. Chioza B. Baple E. L. Maroofian R. Cross H.. (2013). Mutations in B4GALNT1 (GM2 synthase) underlie a new disorder of ganglioside biosynthesis. Brain 136(Pt 12), 3618–3624. 10.1093/brain/awt270 24103911 PMC3859217

[B30] Hellmich H. L. Kos L. Cho E. S. Mahon K. A. Zimmer A. (1996). Embryonic expression of glial cell-line derived neurotrophic factor (GDNF) suggests multiple developmental roles in neural differentiation and epithelial-mesenchymal interactions. Mech. Dev. 54, 95–105. 10.1016/0925-4773(95)00464-5 8808409

[B31] Hidalgo-Figueroa M. Bonilla S. Gutierrez F. Pascual A. Lopez-Barneo J. (2012). GDNF is predominantly expressed in the PV+ neostriatal interneuronal ensemble in normal mouse and after injury of the nigrostriatal pathway. J. Neurosci. 32, 864–872. 10.1523/JNEUROSCI.2693-11.2012 22262884 PMC6621168

[B32] Israeli E. Sharon R. (2009). Beta-synuclein occurs *in vivo* in lipid-associated oligomers and forms hetero-oligomers with α-synuclein. J. Neurochem. 108, 465–474. 10.1111/j.1471-4159.2008.05776.x 19012742 PMC2832289

[B33] Jing S. Wen D. Yu Y. Holst P. L. Luo Y. Fang M.. (1996). GDNF-Induced activation of the ret protein tyrosine kinase is mediated by GDNFR-a, a novel receptor for GDNF. Cell 85, 1113–1124. 10.1016/S0092-8674(00)81311-2 8674117

[B34] Johnston L. C. Eberling J. Pivirotto P. Hadaczek P. Federoff H. J. Forsayeth J.. (2009). Clinically relevant effects of convection-enhanced delivery of AAV2-GDNF on the dopaminergic nigrostriatal pathway in aged rhesus monkeys. Hum. Gene Ther. 20, 497–510. 10.1089/hum.2008.137 19203243 PMC2767387

[B35] Kells A. P. Eberling J. Su X. Pivirotto P. Bringas J. Hadaczek P.. (2010). Regeneration of the MPTP-lesioned dopaminergic system after convection-enhanced delivery of AAV2-GDNF. J. Neurosci. 30, 9567–9577. 10.1523/JNEUROSCI.0942-10.2010 20631185 PMC2914692

[B36] Kordower J. H. Olanow C. W. Dodiya H. B. Chu Y. Beach T. G. Adler C. H.. (2013). Disease duration and the integrity of the nigrostriatal system in Parkinson's disease. Brain 136(Pt 8), 2419–2431. 10.1093/brain/awt192 23884810 PMC3722357

[B37] Kozlowski D. A. Connor B. Tillerson J. L. Schallert T. Bohn M. C. (2000). Delivery of a GDNF gene into the substantia nigra after a progressive 6-OHDA lesion maintains functional nigrostriatal connections. Exp. Neurol. 166, 1–15. 10.1006/exnr.2000.7463 11031079

[B38] Lang A. E. Gill S. Patel N. K. Lozano A. Nutt J. G. Penn R.. (2006). Randomized controlled trial of intraputamenal glial cell line-derived neurotrophic factor infusion in Parkinson disease. Ann. Neurol. 59, 459–466. 10.1002/ana.20737 16429411

[B39] Lindgren N. Francardo V. Quintino L. Lundberg C. Cenci M. A. (2012). A model of GDNF gene therapy in mice with 6-Hydroxydopamine lesions: time course of neurorestorative effects and ERK1/2 activation. J. Parkinsons Dis. 2, 333–348. 10.3233/JPD-012146 23938263

[B40] Lingwood D. Simons K. (2010). Lipid rafts as a membrane-organizing principle. Science 327, 46–50. 10.1126/science.1174621 20044567

[B41] Lonka-Nevalaita L. Lume M. Leppanen S. Jokitalo E. Peranen J. Saarma M. (2010). Characterization of the intracellular localization, processing, and secretion of two glial cell line-derived neurotrophic factor splice isoforms. J. Neurosci. 30, 11403–11413. 10.1523/JNEUROSCI.5888-09.2010 20739562 PMC6633335

[B42] Mahul-Mellier A. L. Fauvet B. Gysbers A. Dikiy I. Oueslati A. Georgeon S.. (2014). c-Abl phosphorylates α-synuclein and regulates its degradation: implication for α-synuclein clearance and contribution to the pathogenesis of Parkinson's disease. Hum. Mol. Genet. 23, 2858–2879. 10.1093/hmg/ddt674 24412932 PMC4014189

[B43] Marks W. J. Jr. Bartus R. T. Siffert J. Davis C. S. Lozano A. Boulis N.. (2010). Gene delivery of AAV2-neurturin for Parkinson's disease: a double-blind, randomised, controlled trial. Lancet Neurol. 9, 1164–1172. 10.1016/S1474-4422(10)70254-4 20970382

[B44] Martinez Z. Zhu M. Han S. Fink A. L. (2007). GM1 specifically interacts with α-synuclein and inhibits fibrillation. Biochemistry 46, 1868–1877. 10.1021/bi061749a 17253773

[B45] Masliah E. Rockenstein E. Veinbergs I. Mallory M. Hashimoto M. Takeda A.. (2000). Dopaminergic loss and inclusion body formation in α-synuclein mice: implications for neurodegenerative disorders. Science 287, 1265–1269. 10.1126/science.287.5456.1265 10678833

[B46] Mazzulli J. R. Xu Y.-H. Sun Y. Knight A. L. McLean P. J. Caldwell G. A.. (2011). Gaucher disease glucocerebrosidase and α-synuclein form a bidirectional pathogenic loop in synucleinopathies. Cell 146, 37–52. 10.1016/j.cell.2011.06.001 21700325 PMC3132082

[B47] Mukherjee A. Arnaud L. Cooper J. A. (2003). Lipid-dependent recruitment of neuronal Src to lipid rafts in the brain. J. Biol. Chem. 278, 40806–40814. 10.1074/jbc.M306440200 12912979

[B48] Mure H. Hirano S. Tang C. C. Isaias I. U. Antonini A. Ma Y.. (2011). Parkinson's disease tremor-related metabolic network: characterization, progression, and treatment effects. Neuroimage 54, 1244–1253. 10.1016/j.neuroimage.2010.09.028 20851193 PMC2997135

[B49] Nakamura T. Yamashita H. Nagano Y. Takahashi T. Avraham S. Avraham H.. (2002). Activation of Pyk2/RAFTK induces tyrosine phosphorylation of α-synuclein via Src-family kinases. FEBS Lett. 521, 190–194. 10.1016/S0014-5793(02)02861-2 12096713

[B50] Okochi M. Walter J. Koyama A. Nakajo S. Baba M. Iwatsubo T.. (2000). Constitutive phosphorylation of the Parkinson's disease associated α-synuclein. J. Biol. Chem. 275, 390–397. 10.1074/jbc.275.1.390 10617630

[B51] Pierchala B. A. Milbrandt J. Johnson E. M. Jr. (2006). Glial cell line-derived neurotrophic factor-dependent recruitment of Ret into lipid rafts enhances signaling by partitioning Ret from proteasome-dependent degradation. J. Neurosci. 26, 2777–2787. 10.1523/JNEUROSCI.3420-05.2006 16525057 PMC6675173

[B52] Plattner R. Kadlec L. DeMali K. A. Kazlauskas A. Pendergast A. M. (1999). c-Abl is activated by growth factors and Src family kinases and has a role in the cellular response to PDGF. Genes Dev. 13, 2400–2411. 10.1101/gad.13.18.2400 10500097 PMC317022

[B53] Polymeropoulos M. H. Lavedan C. Leroy E. Ide S. E. Dehejia A. Dutra A.. (1997). Mutation in the alpha-synuclein gene identified in families with Parkinson's disease. Science 276, 2045–2047. 10.1126/science.276.5321.2045 9197268

[B54] Poteryaev D. Titievsky A. Sun Y. F. Thomas-Crusells J. Lindahl M. Billaud M.. (1999). GDNF triggers a novel ret-independent Src kinase family-coupled signaling via a GPI-linked GDNF receptor alpha1. FEBS Lett. 463, 63–66. 10.1016/S0014-5793(99)01590-2 10601639

[B55] Recasens A. Ulusoy A. Kahle P. J. Di Monte D. A. Dehay B. (2017). *In vivo* models of alpha-synuclein transmission and propagation. Cell Tissue Res. 10.1007/s00441-017-2730-9. [Epub ahead of print] 29185072

[B56] Richardson R. M. Kells A. P. Rosenbluth K. H. Salegio E. A. Fiandaca M. S. Larson P. S.. (2011). Interventional MRI-guided putaminal delivery of AAV2-GDNF for a planned clinical trial in Parkinson's disease. Mol. Ther. 19, 1048–1057. 10.1038/mt.2011.11 21343917 PMC3129792

[B57] Sanchez-Lanzas R. Alvarez-Castelao B. Bermejo T. Ayuso T. Tunon T. Castano J. G. (2016). Protein degradation in a LAMP-2-deficient B-lymphoblastoid cell line from a patient with Danon disease. Biochim. Biophys. Acta 1862, 1423–1432. 10.1016/j.bbadis.2016.04.014 27130438

[B58] Schneider J. S. Roeltgen D. P. Mancall E. L. Chapas-Crilly J. Rothblat D. S. Tatarian G. T. (1998). Parkinson's disease: improved function with GM1 ganglioside treatment in a randomized placebo-controlled study. Neurology 50, 1630–1636. 10.1212/WNL.50.6.1630 9633704

[B59] Schneider J. S. Roeltgen D. P. Rothblat D. S. Chapas-Crilly J. Seraydarian L. Rao J. (1995). GM1 ganglioside treatment of Parkinson's disease: an open pilot study of safety and efficacy. Neurology 45, 1149–1154. 10.1212/WNL.45.6.1149 7783880

[B60] Schneider J. S. Sendek S. Daskalakis C. Cambi F. (2010). GM1 ganglioside in Parkinson's disease: results of a five year open study. J. Neurol. Sci. 292, 45–51. 10.1016/j.jns.2010.02.009 20206941

[B61] Senard J. M. Brefel-Courbon C. Rascol O. Montastruc J. L. (2001). Orthostatic hypotension in patients with Parkinson's disease: pathophysiology and management. Drugs Aging 18, 495–505. 10.2165/00002512-200118070-00003 11482743

[B62] Sheikh K. A. Sun J. Liu Y. Kawai H. Crawford T. O. Proia R. L.. (1999). Mice lacking complex gangliosides develop Wallerian degeneration and myelination defects. Proc. Natl. Acad. Sci. U.S.A. 96, 7532–7537. 10.1073/pnas.96.13.7532 10377449 PMC22120

[B63] Singleton A. B. Farrer M. Johnson J. Singleton A. Hague S. Kachergus J. (2003). Alpha-Synuclein locus triplication causes Parkinson's disease. Science 302:841 10.1126/science.1090278 14593171

[B64] Stirpe P. Hoffman M. Badiali D. Colosimo C. (2016). Constipation: an emerging risk factor for Parkinson's disease? Eur. J. Neurol. 23, 1606–1613. 10.1111/ene.13082 27444575

[B65] Taguchi Y. V. Liu J. Ruan J. Pacheco J. Zhang X. Abbasi J.. (2017). Glucosylsphingosine promotes alpha-synuclein pathology in mutant GBA-associated Parkinson's disease. J. Neurosci. 37, 9617–9631. 10.1523/JNEUROSCI.1525-17.2017 28847804 PMC5628407

[B66] Tansey M. G. Baloh R. H. Milbrandt J. Johnson J. E. M. (2000). GFRa-mediated localization of RET to lipid rafts is required for effective downstream signaling, differentiation, and neuronal survival. Neuron 25, 611–623. 10.1016/S0896-6273(00)81064-8 10774729

[B67] Terzioglu M. Galter D. (2008). Parkinson's disease: genetic versus toxin-induced rodent models. FEBS J. 275, 1384–1391. 10.1111/j.1742-4658.2008.06302.x 18279376

[B68] Treanor J. J. S. Goodman L. de Sauvage F. Stone D. M. Poulsen K. T. Beck C. D.. (1996). Characterization of a multicomponent receptor for GDNF. Nature 382, 80–83. 10.1038/382080a0 8657309

[B69] Wu G. Lu Z. H. Kulkarni N. Amin R. Ledeen R. W. (2011). Mice lacking major brain gangliosides develop parkinsonism. Neurochem. Res. 36, 1706–1714. 10.1007/s11064-011-0437-y 21399908 PMC3155038

[B70] Wu G. Lu Z. H. Kulkarni N. Ledeen R. W. (2012). Deficiency of ganglioside GM1 correlates with Parkinson's disease in mice and humans. J. Neurosci. Res. 90, 1997–2008. 10.1002/jnr.23090 22714832

[B71] Yahi N. Fantini J. (2014). Deciphering the glycolipid code of Alzheimer's and Parkinson's amyloid proteins allowed the creation of a universal ganglioside-binding peptide. PLoS ONE 9:e104751. 10.1371/journal.pone.0104751 25140899 PMC4139322

[B72] Zabrocki P. Bastiaens I. Delay C. Bammens T. Ghillebert R. Pellens K.. (2008). Phosphorylation, lipid raft interaction and traffic of alpha-synuclein in a yeast model for Parkinson. Biochim. Biophys. Acta 1783, 1767–1780. 10.1016/j.bbamcr.2008.06.010 18634833

[B73] Zhang W. Wang T. Pei Z. Miller D. S. Wu X. Block M. L.. (2005). Aggregated alpha-synuclein activates microglia: a process leading to disease progression in Parkinson's disease. FASEB J. 19, 533–542. 10.1096/fj.04-2751com 15791003

[B74] Zhao J. Singleton P. A. Brown M. E. Dudek S. M. Garcia J. G. (2009). Phosphotyrosine protein dynamics in cell membrane rafts of sphingosine-1-phosphate-stimulated human endothelium: role in barrier enhancement. Cell Signal. 21, 1945–1960. 10.1016/j.cellsig.2009.09.002 19755153 PMC3758232

